# N95 filtering facepiece respirator fit assessment outcomes by gender, age, race, and facial hair in a community population sample

**DOI:** 10.1093/annweh/wxag050

**Published:** 2026-06-23

**Authors:** Majid Bagheri Hosseinabadi, Minji Yu, Ashley Petersen, Linsey Griffin, William Durfee, Susan Arnold

**Affiliations:** Division of Environmental Health Sciences, School of Public Health, University of Minnesota, Minneapolis, MN 55455, United States; Department of Clothing and Textiles, College of Human Ecology, Yonsei University, Seoul 03722, Korea; Division of Biostatistics and Health Data Science, School of Public Health, University of Minnesota, Minneapolis, MN 55455, United States; College of Design, University of Minnesota, 1985 Buford Avenue, 240 McNeal Hall, Saint Paul, MN 55108, United States; Department of Mechanical Engineering, College of Science and Engineering, University of Minnesota, Minneapolis, MN 55455, United States; Division of Environmental Health Sciences, School of Public Health, University of Minnesota, Minneapolis, MN 55455, United States

**Keywords:** age group, TSI PortaCount Pro+, quantitative fit test, N95 respirator

## Abstract

The necessity for respiratory protection has increased due to global threats such as pandemics, industrial accidents, and extreme weather events. The effectiveness of N95 filtering facepiece respirators depends on how well they fit the user's face and seal sufficiently. The objective of this study was to assess the critical issue of N95 filtering facepiece respirator fit test pass rates in the general population, specifically examining the influences of gender, age group, and race.

*Methods* This cross-sectional study was conducted with Minnesota State Fair attendees in 2021 and 2022. Participants were asked to complete a demographic questionnaire, which included details on age, gender, race/ethnicity, and facial hair. A quantitative single-exercise fit assessment of N95 filtering facepiece respirators was conducted using a TSI PortaCount Pro+ (Model 8038). The assessment was based on the talking exercise derived from the Occupational Safety and Health Administration respiratory protection protocol, with a criterion of a fit factor ≥100 for passing the fit assessment. Descriptive statistics and multiple logistic regression analyses were performed using RStudio (version 4.4.2) to assess the influence of demographic variables on respirator fit assessment results.

*Results* A total of 384 participants were enrolled, with 63.8% being female and 80.2% identifying as White. Respirator fit assessment pass rates were significantly lower for males with facial hair compared to females and males without facial hair. None of the participants with full beards achieved an acceptable fit. Participants aged 19 yrs or younger had the highest pass rates. Females were 83% more likely to pass the fit assessment compared with males without facial hair (odds ratio [OR] = 1.83, 95% CI: 1.02 to 3.38), while males with facial hair were 68% less likely to pass the fit assessment than males without facial hair (OR = 0.32, 95% CI: 0.13 to 0.73). Nonlinear age-related differences in fit assessment results were observed, with the lowest odds of passing among participants in their 30 and 40 s compared with those aged 19 yrs or younger. No significant differences in the fit assessment pass rates between racial and ethnic groups were observed.

*Conclusion* Gender, age, and facial hair type were found to significantly affect the likelihood of participants passing the fit assessment for N95 filtering facepiece respirators. No significant disparities were observed among racial and ethnic groups. These findings underscore the importance of considering gender, age, and facial hair when developing public health guidelines for respirator protection.

What's Important About This Paper? The general public increasingly wears N95 filtering facepiece respirators to protect against airborne particles, but the quality of respirator fit may differ for this population from that among workers. Using a simplified quantitative fit test, this study found gender, age and facial hair type affected the likelihood of passing the fit test, but no differences were observed among racial and ethnic groups. These findings can support public health guidelines for respirator use.

## Introduction

The current and emerging global threats of natural disasters, including hurricanes and wildfires, have amplified the need for respiratory protection for people of all ages, in both occupational and nonoccupational settings. During environmental disasters and industrial accidents, first responders and community members can be exposed to noninfectious airborne particles such as mold after floods and hurricanes (Cummings et al. 2006, 2007), ash from wildfire and volcanic activities (Steinle et al. 2018; Kelly 2020; Wagner et al. 2022), and air pollutants from chemical spills (McDonald et al. 2020), requiring respiratory protection. The COVID-19 pandemic has also highlighted the critical role of respirators in controlling airborne exposures from infectious human sources. Wearing a well-fitted filtering facepiece respirator (FFR), such as N95 respirators, is considered a common measure for preventing airborne transmission of infectious viruses such as influenza, tuberculosis, severe acute respiratory syndrome (SARS), and other airborne-transmitted diseases in healthcare and community settings (Khairul Hasni et al. 2023; Ather et al. 2026). The Centers for Disease Control and Prevention recommends that healthcare personnel wear appropriate personal protective equipment, including a fit-tested N95 or higher respirator during aerosol-generating procedures involving patients with suspected or confirmed airborne infections (Centers for Disease Control and Prevention 2023). A well-fitted N95 FFR also protects against inhalation exposure to hazards beyond infectious agents.

Although the importance of wearing N95 FFRs to prevent exposure to infectious and noninfectious airborne particles is apparent, the effectiveness of these FFRs in reducing exposure to airborne particles varies from person to person. Effectiveness depends on how well the FFR fits the user's face and seals sufficiently (Kleinjohann and Lange 2020; 3M, 2026; no date). Any air leakage around the perimeter of the respirator due to loss of seal or improper fitting can significantly compromise the respirator's performance and put the wearer at higher risk of exposure to airborne particles (Oestenstad and Bartolucci 2010; Viola et al. 2021; Khairul Hasni et al. 2023). It is reported that virus counts recovered from the skin under an N95 FFR worn without fit testing were not significantly lower than the no-respirator control, while only the N95 FFR worn after fit testing provided considerable virus count reduction compared with the no-respirator condition (Landry et al. 2022).

A key step in a respiratory protection program is respirator fit testing, which can be conducted using either qualitative or quantitative methods. Qualitative fit testing is a pass/fail method, identifying leaks based on whether wearers detect the sweet taste of saccharin solution or the bitter taste of Bitrex introduced around the respirator (Fakherpour et al. 2019). Although inexpensive and easy to perform, these tests provide only a subjective measure of the respirator seal and rely on a healthy olfactory system. However, losing the senses of taste and smell is a widespread disorder, affecting up to 20% of the adult population (Rebholz et al. 2020). Furthermore, about 60% of 35.8 million US adults diagnosed with COVID-19 in 2021 reported olfactory or gustatory dysfunction associated with losses of some ability to taste or smell, with 3.7% and 2.6% of patients experiencing no recovery in smell and taste, respectively (Mitchell et al. 2023). Quantitative fit testing objectively assesses respirator fit through several methods, including counting particles inside and outside the respirator, quantifying the leakage into the respirator, and not relying on the wearer's response to the challenge agent (Racz et al. 2018). Fit testing is widely used in workplace settings; however, the suitability of this assessment type for community settings has not been widely studied (Mueller et al. 2018).

The fit of an N95 FFR can be significantly influenced by various factors such as facial dimensions, facial hair, age, gender, race, and respirator brand/model (Fakherpour et al. 2023). The effects of these factors are primarily studied in working populations, while data on general populations are lacking. Furthermore, respirator design has been historically informed by a relatively narrow demographic that does not reflect the more diverse population and population anthropometric features and thus may not provide sufficient fit for a significant fraction of the working or general population. The current fit test panels developed by the National Institute for Occupational Safety and Health (NIOSH) are matched with the 2000 US census population which may not reflect today's more diverse population (Zhuang et al. 2007). The demographics of the US population have changed significantly since these panels were established, with greater diversity and related differences in facial features that are likely not adequately accounted for in the existing bivariate panel criteria. Furthermore, these studies recruited participants who were required to wear respirators as part of their work and excluded nonoccupational wearers, who are less familiar with donning and doffing practices.

Facial hair, especially thick beards or stubble, can lead to significant biological and nonbiological particles entering an N95 FFR by disrupting the face seal between the N95 FFR and skin. Wearing an N95 FFR may not provide adequate respiratory protection against exposure to infectious and noninfectious aerosols for people who cannot be clean-shaven for religious, medical, or cultural reasons. Several studies have demonstrated that the presence of facial hair substantially reduces the probability of achieving an adequate fit. For example, Zhang et al. (2020) found that filtering facepiece respirator fit factors were significantly lower in clean-shaven Australian healthcare workers compared with those who were not clean-shaven (Zhang et al. 2023). Furthermore, Sandaradura et al. (2020) reported that an increase in facial hair length was related to a reduced likelihood of passing an N95 FFR fit test (Sandaradura et al. 2020). Since federal regulations such as the Occupational Safety and Health Administration (OSHA) Respiratory Protection Standard require wearers to be clean-shaven, quantitative data on the impact of facial hair on respirator fit are scarce. In terms of demographic variables, Milosevic et al. (2022) found that older age and male gender were associated with significantly higher failure rates of quantitative fit testing, and a large proportion (45%) of Australian healthcare workers needed to test on multiple N95 FFR models (Milosevic et al. 2022). Furthermore, facial dimensions such as nose breadth, face width, and chin length have been shown to vary across racial and ethnic groups, which can affect respirator fit and seal integrity (Hobbs-Murphy et al. 2025). Failure to account for these anthropometric differences may contribute to disparities in protection among underrepresented populations.

This study was designed to investigate the performance of a quantitative single-exercise fit assessment for a commonly used N95 FFR (3M Aura Respirator 9205+) in a sample of individuals from the general population. Furthermore, this study aimed to consider the role of gender, age, race/ethnicity, and facial hair in the fit assessment pass rate. Unlike prior studies conducted primarily in occupational settings, this study evaluated respirator fit in a public setting, providing insight into respirator performance among untrained members of the general population. This new knowledge will help inform public health guidance during respiratory disease outbreaks.

## Methods

### Study population and participant recruitment

In this cross-sectional study, the target population was the general population attending the Minnesota State Fair in 2021 (from 26 August to 6 September) and 2022 (from 25 August to 5 September). The inclusion criterion was people older than 5 yrs. For participants under the age of 18, parental or guardian consent was obtained before their child was accepted into the study. The study participants constituted a convenience sampling of people who were available during the State Fair and willing to participate in this study. The study was conducted in the University of Minnesota Driven to Discover (D2D) building located on the grounds of the State Fair. Institutional Review Board approval was obtained prior to the start of the study (IRB STUDY00012114). We conducted a priori power analysis to estimate the required sample size for detecting gender differences in N95 FFR fit test pass rates, assuming a 20% effect size based on limited available data in the general population. The analysis determined that at least 161 participants were needed to achieve 80% power at a 0.05 significance level.

The informed consent process started with verbal confirmation by prospective participants that they were vaccinated against COVID-19. The researcher then described the study and showed the prospective participants the measurement tools and the fit assessment instrument. Participants were given the opportunity to read through the consent form. An assent information sheet was provided to parents of children ages 12 to 15, and consent was provided by one parent, digitally. A digital signature and permissions were obtained for all participants via Qualtrics using an iPad.

### Data collection

#### Demographic questionnaire

The research staff greeted potential participants who entered the D2D building and invited them to participate in the study after explaining the study objectives and eligibility criteria. The research staff asked them to complete a demographic questionnaire. The research staff were available to answer any further questions about the study and provide more explanations on the questionnaire. The participants were not asked for personal identifying information. Demographic information included age (in years), gender, race/ethnicity, and facial hair (yes/no and type). The different types of facial hair were goatee, stubble, mustache, and full beard. In addition to the self-reported information on the demographic questionnaire, the research staff also reported their observations on the participants’ type of facial hair.

#### Quantitative fit test

A quantitative fit assessment of N95 FFRs (3M Aura Respirator 9205+) was performed using a TSI PortaCount Pro+ model 8038 condensation particle counter (TSI, Shoreview, MN, USA). This instrument measures respirator fit factors with an efficiency of more than 99%, by counting particles as small as 0.02 micrometers at a flow rate of 350 mL/min inside and outside of the respirator. Total inward leakage (TIL) is calculated based on the inverse relationship between TIL and the fit factor (Rengasamy et al. 2014). The TSI PortaCount Pro+ was operated in N95 mode using the N95-Companion technology. In this configuration, the maximum reportable fit factor is 200, consistent with the manufacturer's specifications. Before each testing session, ambient aerosol concentration was verified using the PortaCount Daily Checks function, which includes a Particle Check to ensure sufficient ambient particle levels (≥30 particles/cm^3^) for valid quantitative fit testing (TSI 2015).

The 3M Aura 9205+ model was selected for this study because it was widely available during the COVID-19 pandemic and at the time of our study. It is also frequently recommended for public use during health emergencies, and designed to fit a broad range of adult face sizes and to allow for natural facial movements when talking (3M 2026; no date). While it is not explicitly marketed for pediatric use, it was included in this study to explore its potential applicability for a broader population of users, both younger and older than the standard working population (18 to 65 yrs) during emergencies when pediatric-specific models may not be available.

The quantitative single-exercise respirator fit assessment was based on the talking exercise from the quantitative OSHA 29 CFR 1910.134 standard protocol with a criterion of ≥100 for a passing fit factor (Occupational Safety Health Administration 2006). Participants read the Rainbow Passage for 60 s while the fit factor was determined. This decision was based on previous findings indicating that this exercise is particularly sensitive to detecting poor N95 FFR fit compared with other exercises (Occupational Safety Health Administration 2016; Griffin et al. 2022; Yu et al. 2024). Furthermore, instead of the fast-full method for 7.2 min or the fast-half method for 2.5 min (Xu et al. 2023), we aimed to provide an objective assessment of fit within a short period of time that participants would tolerate and collect data from a larger and more diverse sample. Although the OSHA fit testing protocol is primarily designed for occupational settings and adults, this simplified approach was applied uniformly and allowed us to maintain consistency while ensuring feasibility for younger participants in a public environment.

Participants were provided with a 3M Aura 9205+ FFR and shown how to don the respirator, including proper placement of the straps and molding of the nose clip around the nose, without any formal training. They were also not taught how to perform a user seal check. This design allowed us to simulate real-world conditions and evaluate how well untrained individuals from the general population were able to don the respirator and achieve an adequate fit.

#### Statistical analysis

Descriptive statistics were used to summarize demographic information and the distribution of the respirator fit factor across the covariates. Wilson score intervals were used to calculate confidence intervals (CIs) for the proportions. Chi-square tests or Fisher's exact tests were used to compare the fit assessment pass percentage between genders, age groups, racial/ethnic groups, and different types of facial hair. Multiple logistic regression was used to compare the binary pass–fail N95 FFR fit factor between the categories of gender, age groups, and racial/ethnic groups. Interaction terms between age groups and facial hair status (male with facial hair vs. all others), and between race/ethnicity and facial hair status, were evaluated in multivariable logistic regression models using likelihood–ratio tests. All statistical tests were performed using RStudio (version 4.4.2) (R Studio Team 2015), with statistical testing performed at a significance level of 0.05.

## Results

A total of 384 participants were enrolled in the study (Table 1). The majority of participants were female (63.8%). About half of the participants were older than 50, indicating a significant representation of older adults compared to other age groups. Participants in the ≤19 age group ranged from 12 to 19 yrs, with most aged 15 to 17 yrs. Most participants identified as White (80.2%), followed by Asian (7.0%). Among males with facial hair, the most common style was stubble (38.0%), followed by full beard (35.2%). In comparing data collected in 2021 and 2022, the proportion of males with facial hair increased from 13.2% in 2021 to 20.7% in 2022. The age distribution also shifted, with an increase in participants aged 60 to 69 (from 9.6% in 2021 to 25.6% in 2022) and those aged 70+ (from 3.5% in 2021 to 18.9% in 2022). Moreover, there was an increase in the proportion of White participants from 71.9% in 2021 to 83.7% in 2022, while the proportion of American Indian or Alaskan Native participants decreased from 12.3% in 2021 to 0.4% in 2022.

**Table 1:** Demographic information of the study participants by year.

Variable	Category	Frequency (%)		
		2021 (*n* = 114)	2022 (*n* = 270)	Total (*n* = 384)
Gender	Female	74 (64.9)	171 (63.3)	245 (63.8)
	Male with facial hair	15 (13.2)	56 (20.7)	71 (18.5)
	Male without facial hair	23 (20.2)	39 (14.4)	62 (16.1)
	Nonbinary	2 (1.8)	4 (1.5)	6 (1.6)
Age group	≤19	27 (23.7)	33 (12.2)	60 (15.6)
	20 to 29	21 (18.4)	22 (8.1)	43 (11.2)
	30 to 39	14 (12.3)	27 (10.0)	41 (10.7)
	40 to 49	13 (11.4)	28 (10.4)	41 (10.7)
	50 to 59	24 (21.1)	40 (14.8)	64 (16.7)
	60 to 69	11 (9.6)	69 (25.6)	80 (20.8)
	70+	4 (3.5)	51 (18.9)	55 (14.3)
Race and ethnicity	White	82 (71.9)	226 (83.7)	308 (80.2)
	American Indian or Alaskan Native	14 (12.3)	1 (0.4)	15 (3.9)
	Asian	3 (2.6)	24 (8.9)	27 (7.0)
	Black or African American	3 (2.6)	9 (3.3)	12 (3.1)
	Hispanic or Latino	8 (7.0)	8 (3.0)	16 (4.2)
	Other	4 (3.5)	2 (0.7)	6 (1.6)
Facial hair (males with facial hair only)^a^	Full beard	4 (26.7)	21 (37.5)	25 (35.2)
	Goatee	3 (20.0)	8 (14.3)	11 (15.5)
	Mustache	0 (0.0)	8 (14.3)	8 (11.3)
	Stubble	8 (53.3)	19 (33.9)	27 (38.0)

^a^Facial hairstyle was assessed only among males with facial hair (*n* = 71).

**Figure 1:**
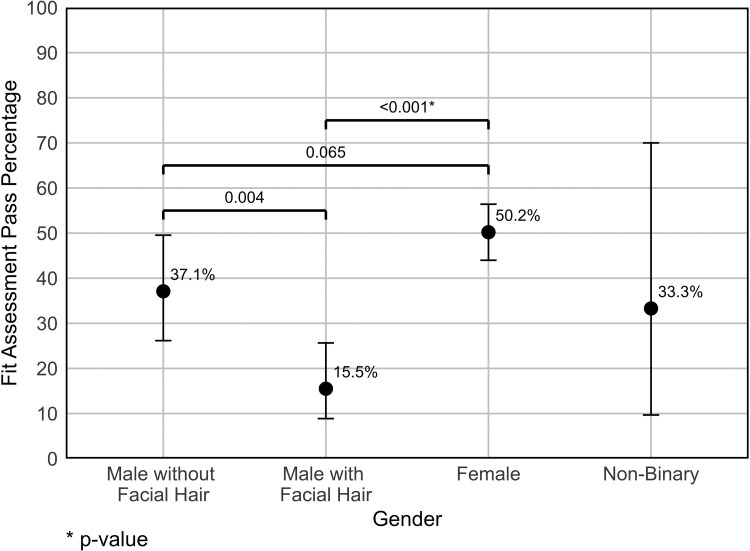
Fit assessment pass rates (%) by gender, with 95% CI.

**Figure 2:**
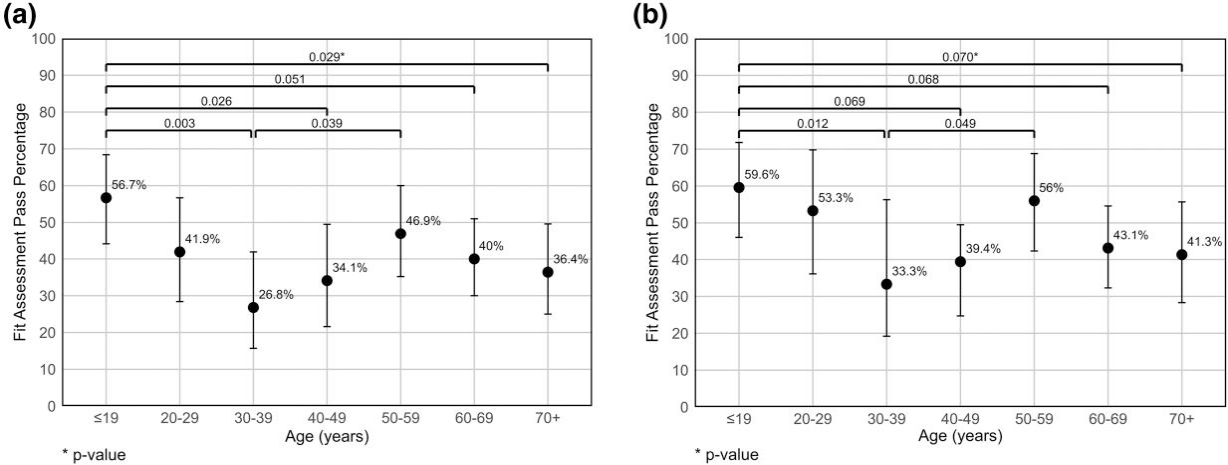
Fit assessment pass rates (%) by age group, including (left) and excluding (right) males with facial hair, with 95% CI.

**Figure 3:**
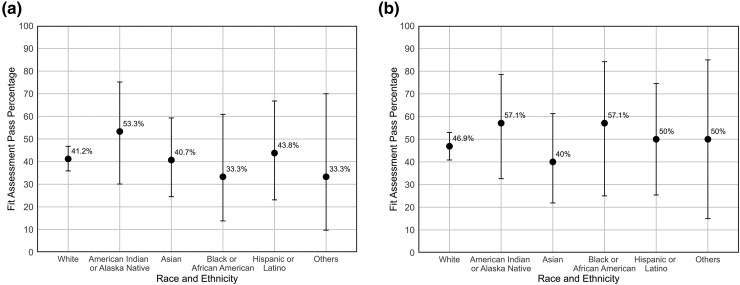
Fit assessment pass rates (%) by racial/ethnic group, including (left) and excluding (right) males with facial hair, with 95% CI (no significant differences observed).

**Figure 4:**
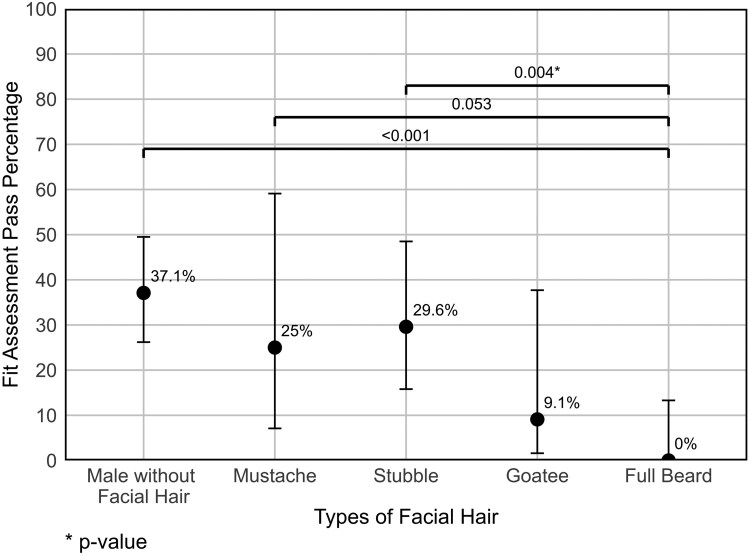
Fit assessment pass rates (%) by the type of facial hair, with 95% CI.

The fit assessment pass rate of males with facial hair was significantly lower than that of males without facial hair (15.5% vs. 37.1%, *P* = 0.004) and females (15.5% vs. 50.2%, *P* < 0.001), as shown in Fig. 1. Furthermore, the difference between the fit assessment pass rate of males without facial hair and females approached significance (37.1% vs. 50.2%, *P* = 0.065). The fit assessment pass rate of participants who were 19 yrs old or younger was 56.7%, significantly higher than the fit assessment pass rate of participants in the 30 to 39, 40 to 49, 60 to 69, and 70+ age groups, even after excluding males with facial hair (Fig. 2). There was a significant difference between the fit assessment pass rate of participants in the 30 to 39 and 50 to 59 age groups after excluding males with facial hair. There were no significant differences in the fit assessment pass rate between racial/ethnic categories when including and excluding males with facial hair (Fig. 3). None of the participants with full beards passed the fit assessment. There was no significant difference in the fit assessment pass rate between participants with goatees and full beards (Fig. 4).

Females had a more even distribution across all fit factor score categories, with the highest percentage (20.8%) achieving a fit factor between 100 and 124. In contrast, males with facial hair showed a high percentage (26.8%) in the <25 and 50 to 74 fit factor score categories, indicating poorer fit overall (Appendix, Table S1). The age group ≤19 yrs had 30.0% achieving a fit factor of ≥150, the highest among all age groups. The 60 to 69 age group had a significant portion (25.0%) in the 50 to 74 category, showing varied fit performance. White participants had a balanced distribution with peaks (18.2%) in the 75 to 99 and 100 to 124 fit factor score categories. Hispanic or Latino participants had 31.3% achieving a fit factor of ≥150, the highest among all ethnic groups. Males with a full beard showed 52.0% with a fit factor <25, indicating poor fit. Males with stubble had a more varied distribution, with 33.3% achieving a fit factor between 50 and 74.

**Figure 5:**
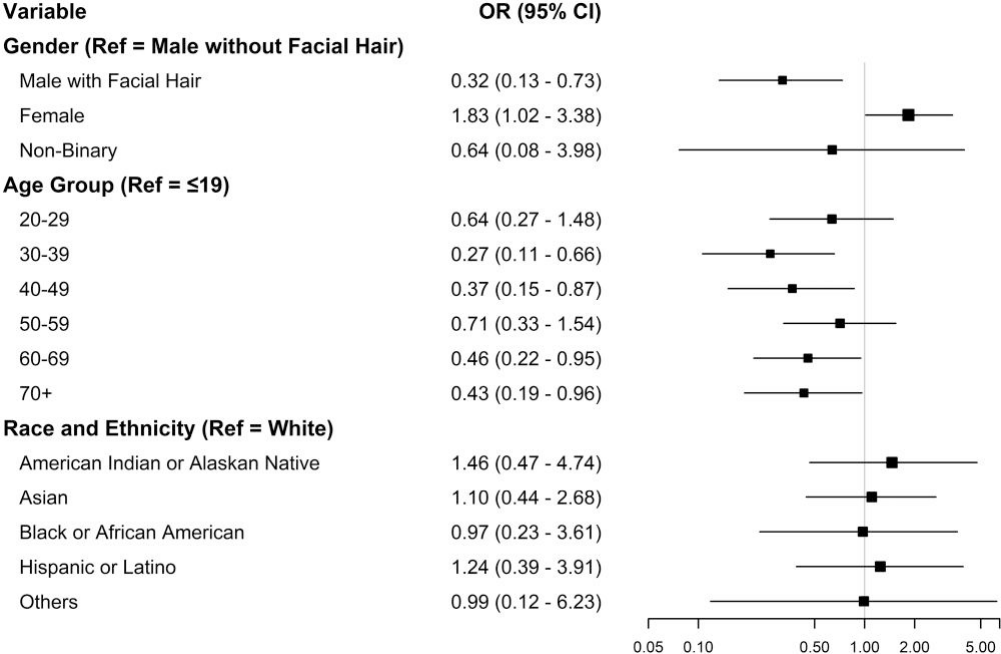
Adjusted ORs of passing the fit assessment by gender, age group, and racial/ethnic group.

Females had significantly higher odds (OR = 1.83, 95% CI: 1.02 to 3.38) of passing the fit assessment compared to males without facial hair (Fig. 5). Males with facial hair had significantly lower odds (OR = 0.32, 95% CI: 0.13 to 0.73) of passing the fit assessment than males without facial hair. Participants aged 30 to 39 had significantly lower odds (OR = 0.27, 95% CI: 0.11 to 0.66) of passing the fit assessment than those under 19. Similarly, the 40 to 49 (OR = 0.37, 95% CI: 0.15 to 0.87), 60 to 69 (OR = 0.46, 95% CI: 0.22 to 0.95), and 70+ (OR = 0.43, 95% CI: 0.19 to 0.96) age groups also had significantly lower odds of passing the fit assessment. None of the non-White racial/ethnic groups showed statistically significant differences in odds of passing the fit assessment compared to White participants.

Analysis of interactions showed no evidence of interactions between age group and facial hair status (*P* = 0.988) and between race/ethnicity and facial hair status (*P* = 0.402).

## Discussion

This study evaluated a quantitative single-exercise respirator fit assessment for the 3M Aura model 9205 N95™ FFR, following the talking exercise from the OSHA 29 CFR 1910.134 respiratory protection protocol, using the Rainbow Passage reading component. Study participants were Minnesota State Fair attendees in 2021 and 2022 and spanned ages 12 to 88 yrs. Factors examined were gender, age, race/ethnicity, and facial hair. Guidance and regulations from government agencies mainly focus on fit testing respirators among working populations, while there is a lack of information about the fit of N95 FFRs in the general population. Furthermore, there are few studies on the role of demographic factors in passing fit assessment outcomes in the general population. These findings extend prior occupational fit-testing research by demonstrating respirator fit performance in a general population setting, where prior training and respirator familiarity may vary.

### Gender and fit assessment pass rates

Gender was a significant factor in fit assessment outcomes for this respirator model, with more females passing compared to males, especially those with facial hair. Nonbinary individuals showed no statistical difference, though the small sample size limits conclusions. Males with facial hair had notably lower odds of passing, while females had higher odds compared to males without facial hair, indicating better alignment between design and anthropometry for females than for males.

The results of previous studies of the effect of gender on passing fit tests are mixed, but most of these studies are among working populations such as healthcare workers. Variations in facial dimensions, respirator designs, and the specific populations studied highly influence the results of gender differences in the fit test pass rate. A recent systematic review by Fakherpour et al. (2023) found that males’ fit test pass rates were higher than females’ in four studies (Fakherpour et al. 2023). However, three studies reported higher fit test pass rates for females than males, and two studies found no significant association between fit test pass rates and gender (Green et al. 2021; Seo et al. 2021; Milosevic et al. 2022). All of these studies were conducted among healthcare workers. Zhang et al. (2020) studied the association of anthropometric measurements of 87 Chinese males and females with FFR fit test outcomes and reported no significant difference in fit test results. They mentioned that the differences in facial characteristics between males and females do not affect the results of the fit test pass rate (Zhang et al. 2020).

However, Yu et al. (2014) found that Chinese males had a higher fit test pass rate for the cup respirators, while Chinese females had a higher rate of passing the fit test for the folding respirators (Yu et al. 2014). Khairul Hasni et al. (2023) studied six different N95 FFR designs across gender and facial dimensions in 135 Malaysians. They reported that males had higher rates of passing the fit test for Trifold A and B compared with Malaysian females (62.0% vs. 41.5% and 79.3% vs. 70.1%), which might be due to a better fit of those respirators with males’ larger facial sizes. In comparison, females’ smaller facial sizes fit better with Cup A and Duckbill B respirators (57.1% vs. 56.8% and 84.4% vs. 82.7%) (Khairul Hasni et al. 2023). In a study by Milosevic et al. (2022) among 11,550 Australian healthcare workers, males were 15% less likely (OR = 0.85, 95% CI: 0.78 to 0.93, adjusted for age and FFR model) to pass fit testing compared with their female counterparts. Males with more than 1 d of facial hair growth were excluded from the study. They argued that one reason for the higher probability of females passing the fit test may be the availability of smaller versions of FFRs across all tested models (Milosevic et al. 2022).

Overall, there is a lack of studies on the performance of nonbinary participants in fit test pass rates (Brisbine et al. 2022). Some studies excluded them from analysis because they represented a very small proportion of the total sample size (Wang et al. 2024). For instance, Caggiari et al. (2023) excluded nonbinary participants from their analysis as they were only 0.1% of the total cohort (Caggiari et al. 2023).

### Age group and fit assessment pass rates

Significant, nonlinear age-related differences in fit assessment results were observed, with the highest pass rates among participants aged 19 yrs or younger, followed by a decline through middle age, a slight recovery in those aged 50 to 59, and another decrease in older participants. Excluding males with facial hair increased pass rates across all age groups. The youngest group showed the best fit assessment performance, suggesting better protection for this age group, while participants in their 30 and 40 s had the lowest odds of passing. No significant differences were found for those in their 20 and 50 s compared to the youngest group.

The results of previous studies on the role of age in fit test pass rates are inconsistent. In their systematic review, Fakherpour et al. (2023) found only two papers considering the impact of age group on fit test pass rates among healthcare workers (Fakherpour et al. 2023). Similar to our results, Milosevic et al. (2022) reported a nonlinear, decreasing trend in the fit test pass rate of Australian healthcare workers in age groups 30 to 39 (OR = 0.79, 95% CI: 0.70 to 0.88), 40 to 49 (OR = 0.77, 95% CI: 0.68 to 0.88) and 50 to 59 (OR = 0.79, 95% CI: 0.69 to 0.91) compared with the age group of 18 to 29, adjusted for age and FFR models. However, there was no significant difference in fit test pass rates between the age groups 18 to 29 and 60+ (OR = 0.88, 95% CI: 0.77 to 1.01) (Milosevic et al. 2022). Furthermore, Zhang et al. (2023) found that healthcare workers in the 18 to 29 age group had a higher fit test pass rate for three-panel flat fold respirators (3M Aura 1870+, 3M Aura 9320a, and Industree Trident) compared to those in the 30 to 39, 40 to 49, 50 to 59, and 60+ age groups. They showed a 1% to 2% decrease in the odds of passing fit tests for these respirators with each 1-yr increase in age, adjusted for gender, clean-shaven status, and fit tester (Zhang et al. 2023).

Nevertheless, Park et al. (2021) reported a 2% increase in the odds of passing the fit test in 30 South Korean healthcare workers with each additional year of age, adjusting for gender, facial width and length, body mass index, occupation, department of working, career history, and education in respiratory usage (Park et al. 2021).

### Race/ethnicity and fit assessment pass rates

No significant differences in performance associated with race/ethnicity were found. While participants represented multiple races/ethnicities, most participants were White. Consequently, the implications of the impact of race/ethnicity on fit are limited by the study's relatively homogeneous demographic makeup. Similar to our study, Manganyi et al. (2017) reported no significant association between race and the fit factor of N95 FFRs in their multiple logistic regression analysis among 562 employees in six laboratories handling samples for tuberculosis testing in Gauteng Province, South Africa. They argued that race might be associated with more proximal factors such as face length and nose bridge width (Manganyi et al. 2017). A systematic review and meta-analysis of healthcare workers by Chopra et al. (2021) revealed that smaller facial measurements in females are not always associated with lower fit factor scores despite significant differences in anthropometric measurements between ethnicities. The comparison between different races in respirator fit was not established due to underrepresented minority ethnicities in these studies and considerable heterogeneity (Chopra et al. 2021).

In contrast to our study's results in the general population, Caggiari et al. (2023) and Carvalho et al. (2021) found that White healthcare workers in English hospitals were about 40% (adjusted for gender, type of test, and body mass index) and 50% (adjusted for gender and FFR model) more likely to pass the fit test compared with Asian, Black, and Mixed/multiple-background healthcare workers (Carvalho et al. 2021; Caggiari et al. 2023). Moreover, Sheikh et al. (2023) found that non-White Canadian healthcare workers had lower mean fit test scores compared with White Canadian healthcare workers (Sheikh et al. 2023).

### Facial hair type and fit assessment pass rates

The results showed significant differences in respirator fit based on facial hair type, with a progressive decrement in fit assessment pass rates as the area of skin covered by facial hair and the length of facial hair increased. Men without facial hair had the highest pass rates, followed by those with stubble, mustaches, and goatees. No males with full beards were able to pass the N95 FFR fit assessment. In one of the few studies in the general public, Prince et al. (2021) found a linear decrease in the fit of NIOSH-approved N95 FFR and Korean standard KF94 in ten adult male staff with increasing beard length within the range of 0 to 10 mm; this decrease was more variable for the Chinese standard KN95. They also found that using an elastic exercise band to cover beard hair significantly improved the fit of face masks (Prince et al. 2021). Sandaradura et al. (2020) found that the fit test pass rate among clean-shaven male hospital employees was 47% (*n* = 38), which is higher than our results. However, similar to our results, none of the males with full beards (*n* = 20) passed the fit test. They also found that a longer length of facial hair is related to a lower likelihood of passing the fit test; passing the fit test in light stubble and heavy stubble were 40% (*n* = 20) and 29% (*n* = 21), respectively. The odds of passing the fit test in males with full beards were 96% lower (OR = 0.04, 95% CI: 0.0 to 0.28) than in males with no facial hair (Sandaradura et al. 2020).

### Limitations

This study has several limitations. We tested fit for only one model of FFR (3M Aura 9205+), which was selected due to its wide availability and adaptable design. However, it is not specifically designed for children, and its performance in pediatric populations may not reflect that of child-specific models. We used only one of the components of the OSHA fit testing protocol to reduce participant burden and ensure the feasibility of fit testing in younger participants. Although this component has been shown to effectively detect poor fit, excluding the other exercises may have resulted in the under detection of some fit failures. Furthermore, participants were recruited from among the Minnesota State Fair attendees, which may introduce selection bias. State Fair attendees may not represent the broader US general population in terms of socioeconomic status, geographic diversity, or health-related behaviors. As such, our findings may not be fully generalizable to the general population. We did not collect information on prior respirator training, fit testing history, or familiarity with respiratory protection. It is possible that higher fit assessment pass rates observed in some participants were influenced by better donning practices rather than an inherently better seal to the face. These unmeasured factors may have influenced fit assessment performance and represent potential sources of unmeasured confounding. While no significant differences in fit assessment pass rates were observed across racial and ethnic groups, this finding should be interpreted with caution. A post hoc power analysis showed that several subgroups did not meet the sample size needed to detect moderate differences, suggesting the study may have been underpowered for these comparisons. Additional studies that include a range of FFR models using the complete OSHA fit testing protocol, administered to volunteers from more representative samples and a broader range of races/ethnicities, are needed to support respiratory protection guidance in cases of public health emergencies. These data would also inform respirator design to improve fit across a broader range of wearers.

## Conclusions

Significant differences in the odds of participants passing the fit assessment were observed by gender, age, and facial hair type. Notably, none of the male participants with full beards passed the quantitative fit assessment. However, there were no significant differences in the fit assessment pass rates between racial and ethnic groups in this moderately diverse population. These results suggest that public health guidance related to FFR use should account for gender-, age-, and facial hair-related differences in fit performance.

## Supplementary Material

wxag050_Supplementary_Data

## Data Availability

Data used in this study are available from the Data Repository for the University of Minnesota (DRUM). https://doi.org/10.13020/aw5t-0a47.

## References

[wxag050-B1] 3M . 2026. 3M Aura particulate respirator 9205+. https://www.3m.com/3M/en_US/p/d/b00051022/. Accessed February 12, 2026.

[wxag050-B2] 3M . no date. 3M Center for respiratory protection. https://www.3m.com/3M/en_US/respiratory-protection-us/support/center-for-respiratory-protection/fit-testing/. Accessed February 12, 2026.

[wxag050-B3] Ather B, Mirza TM, Edemekong PF. 2026. Airborne precautions. https://www.ncbi.nlm.nih.gov/books/NBK531468/. Accessed February 12, 2026. 30285363

[wxag050-B4] Brisbine BR, Radcliffe CR, Jones ML, Stirling L, Coltman CE. 2022. Does the fit of personal protective equipment affect functional performance? A systematic review across occupational domains. PLoS One. 17:e0278174. 10.1371/journal.pone.0278174. 36449531 PMC9710848

[wxag050-B5] Caggiari S et al 2023. Retrospective evaluation of factors affecting successful fit testing of respiratory protective equipment during the early phase of COVID-19. BMJ Open. 13:e065068. 10.1136/bmjopen-2022-065068. PMC10230346 37230519

[wxag050-B6] Carvalho CY, Schumacher J, Greig PR, Wong DJ, El-Boghdadly K. 2021. Prospective observational study of gender and ethnicity biases in respiratory protective equipment for healthcare workers in the COVID-19 pandemic. BMJ Open. 11:e047716. 10.1136/bmjopen-2020-047716. PMC8141377 34016664

[wxag050-B7] Centers for Disease Control and Prevention . 2023. Appendix A: Table 4. Recommendations for Application of Standard Precautions for the Care of All Patients in All Healthcare Settings. https://www.cdc.gov/infection-control/hcp/isolation-precautions/appendix-a-table-4.html. Accessed February 12, 2026.

[wxag050-B8] Chopra J et al 2021. The influence of gender and ethnicity on facemasks and respiratory protective equipment fit: a systematic review and meta-analysis. BMJ Glob Health. 6:e005537. 10.1136/bmjgh-2021-005537. PMC8587533 34764145

[wxag050-B9] Cummings KJ et al 2006. Knowledge, attitudes, and practices related to mold exposure among residents and remediation workers in posthurricane New Orleans. Arch Environ Occup Health. 61:101–108. 10.3200/aeoh.61.3.101-108. 17672351

[wxag050-B10] Cummings KJ, Cox-Ganser J, Riggs MA, Edwards N, Kreiss K. 2007. Respirator donning in post-hurricane New Orleans. Emerg Infect Dis. 13:700–707. 10.3201/eid1305.061490. 17553247 PMC2738466

[wxag050-B11] Fakherpour A, Jahangiri M, Jansz J. 2023. A systematic review of passing fit testing of the masks and respirators used during the COVID-19 pandemic: part 1-quantitative fit test procedures. PLoS One. 18:e0293129. 10.1371/journal.pone.0293129. 37883443 PMC10602271

[wxag050-B12] Fakherpour A, Jahangiri M, Yousefinejad S, Seif M. 2019. Feasibility of replacing homemade solutions by commercial products for qualitative fit testing of particulate respirators: a mixed effect logistic regression study. MethodsX. 6:1313–1322. 10.1016/j.mex.2019.05.034. 31205864 PMC6558090

[wxag050-B13] Green S, Gani A, Bailey M, Brown O, Hing C. 2021. Fit-testing of respiratory protective equipment in the UK during the initial response to the COVID-19 pandemic. J Hosp Infect. 113:180–186. 10.1016/j.jhin.2021.04.024. 33940089 PMC8087583

[wxag050-B14] Griffin L et al 2022. Protective masks utilizing nonendangered components. J Med Device. 16:015001. 10.1115/1.4053720. 35280214 PMC8905092

[wxag050-B16] Hobbs-Murphy K, Brazile WJ, Morris K, Rosecrance J. 2025. Demographic differences in facial anthropometric data from 3D scans and implications for respirator fit. Ann Work Expo Health. 69:442–452. 10.1093/annweh/wxaf012. 40106729 PMC12018073

[wxag050-B17] Kelly K . 2020. Informing the use of N95 respirators by the general public to reduce wildfire smoke exposure. University of Washington.

[wxag050-B18] Khairul Hasni NA et al 2023. The effect of N95 designs on respirator fit and its associations with gender and facial dimensions. PLoS One. 18:e0288105. 10.1371/journal.pone.0288105. 38019763 PMC10686483

[wxag050-B19] Kleinjohann L, Lange C. 2020. Respirators evaluated by fit testing. ERJ Open Res. 6:00581-2020. 10.1183/23120541.00581-2020. 33263063 PMC7682718

[wxag050-B20] Landry SA et al 2022. Fit-tested N95 masks combined with portable high-efficiency particulate air filtration can protect against high aerosolized viral loads over prolonged periods at close range. J Infect Dis. 226:199–207. 10.1093/infdis/jiac195. 35535021 PMC9400421

[wxag050-B21] Manganyi J, Wilson KS, Rees D. 2017. Quantitative respirator fit, face sizes, and determinants of fit in South African diagnostic laboratory respirator users. Ann Work Expo Health. 61:1154–1162. 10.1093/annweh/wxx077. 29136414

[wxag050-B22] McDonald F et al 2020. Facemask use for community protection from air pollution disasters: an ethical overview and framework to guide agency decision making. Int J Disaster Risk Reduct. 43:101376. 10.1016/j.ijdrr.2019.101376.

[wxag050-B23] Milosevic M et al 2022. P2/N95 filtering facepiece respirators: results of a large-scale quantitative mask fit testing program in Australian health care workers. Am J Infect Control. 50:509–515. 10.1016/j.ajic.2021.12.016. 34971710 PMC8767955

[wxag050-B24] Mitchell MB, Workman AD, Rathi VK, Bhattacharyya N. 2023. Smell and taste loss associated with COVID-19 infection. Laryngoscope. 133:2357–2361. 10.1002/lary.30802. 37265267

[wxag050-B25] Mueller W et al 2018. The effectiveness of respiratory protection worn by communities to protect from volcanic ash inhalation. Part I: filtration efficiency tests. Int J Hyg Environ Health. 221:967–976. 10.1016/j.ijheh.2018.03.012. 29779694

[wxag050-B26] Occupational Safety Health Administration . 2006. Major requirements of OSHA's Respiratory protection standard 29 CFR 1910.134. OSHA Office of Training and Education.

[wxag050-B27] Occupational Safety Health Administration . 2016. Additional portacount quantitative fit-testing protocols: amendment to respiratory protection standard. Fed Regist. 81:69740–69751.

[wxag050-B28] Oestenstad RK, Bartolucci AA. 2010. Factors affecting the location and shape of face seal leak sites on half-mask respirators. J Occup Environ Hyg. 7:332–341. 10.1080/15459621003729909. 20379896

[wxag050-B29] Park JJ, Seo YB, Lee J. 2021. Fit test for N95 filtering facepiece respirators and KF94 masks for healthcare workers: a prospective single-center simulation study. J Korean Med Sci. 36:e140. 10.3346/jkms.2021.36.e140. 34060256 PMC8167410

[wxag050-B30] Prince SE et al 2021. Assessing the effect of beard hair lengths on face masks used as personal protective equipment during the COVID-19 pandemic. J Expo Sci Environ Epidemiol. 31:953–960. 10.1038/s41370-021-00337-1. 34006963 PMC8130778

[wxag050-B31] Racz L, Yamammoto DP, Eninger RM. 2018. Handbook of respiratory protection safeguarding against current and emerging hazards. CRC Press.

[wxag050-B32] Rebholz H et al 2020. Loss of olfactory function—early indicator for COVID-19, other viral infections and neurodegenerative disorders. Front Neurol. 11:569333. 10.3389/fneur.2020.569333. 33193009 PMC7649754

[wxag050-B33] Rengasamy S et al 2014. Total inward leakage measurement of particulates for N95 filtering facepiece respirators—a comparison study. Ann Occup Hyg. 58:206–216. 10.1093/annhyg/met054. 24107745

[wxag050-B34] R Studio Team . 2015. R Studio: integrated development for R. RStudio, Inc.

[wxag050-B35] Sandaradura I et al 2020. A close shave? Performance of P2/N95 respirators in healthcare workers with facial hair: results of the BEARDS (BEnchmarking adequate respiratory DefenceS) study. J Hosp Infect. 104:529–533. 10.1016/j.jhin.2020.01.006. 31978416

[wxag050-B36] Seo H, Myong J, Kang B, Kwon Y. 2021. Necessity of the fit test panel for Korean respirator users: application to Korean healthcare workers. J Int Soc Respir Prot. 38:1–11.

[wxag050-B37] Sheikh F, Schwartz L, Dolovich M, Fox-Robichaud A. 2023. N95 respirators for a diverse population of healthcare workers: a mixed-methods, prospective, pilot and feasibility study. Springer.

[wxag050-B38] Steinle S et al 2018. The effectiveness of respiratory protection worn by communities to protect from volcanic ash inhalation. Part II: total inward leakage tests. Int J Hyg Environ Health. 221:977–984. 10.1016/j.ijheh.2018.03.011. 29861400

[wxag050-B39] TSI . 2015. Portacount Pro 8030 and PortaCount Pro+ 8038 respirator fit testers operation and service manual. TSI Shoreview.

[wxag050-B40] Viola IM et al 2021. Face coverings, aerosol dispersion and mitigation of virus transmission risk. IEEE Open J Eng Med Biol. 2:26–35. 10.1109/ojemb.2021.3053215. 34812420 PMC8545035

[wxag050-B41] Wagner J, Macher JM, Chen W, Kumagai K. 2022. Comparative mask protection against inhaling wildfire smoke, allergenic bioaerosols, and infectious particles. Int J Environ Res Public Health. 19:15555. 10.3390/ijerph192315555. 36497628 PMC9735667

[wxag050-B42] Wang RC et al 2024. Incidence of fit test failure during N95 respirator reuse and extended use. JAMA Netw Open. 7:e2353631. 10.1001/jamanetworkopen.2023.53631. 38277142 PMC12282505

[wxag050-B43] Xu X et al 2023. Conducting quantitative mask fit tests: application details and affecting factors. Front Public Health. 11:1218191. 10.3389/fpubh.2023.1218191. 37521986 PMC10372483

[wxag050-B44] Yu M, Griffin L, Durfee WK, Arnold S. 2024. Face anthropometry for filtering facepiece respirators: analysis of the association between facial dimensions and respirator fit. Ann Work Expo Health. 68:312–324. 10.1093/annweh/wxae005. 38366891 PMC10941723

[wxag050-B45] Yu Y et al 2014. Fitting characteristics of N95 filtering-facepiece respirators used widely in China. PLoS One. 9:e85299. 10.1371/journal.pone.0085299. 24465528 PMC3897424

[wxag050-B46] Zhang MM et al 2023. Striving to be the fittest: quantitative P2/N95 respirator fit test results among hospital staff during the COVID-19 pandemic. Antimicrob Steward Healthc Epidemiol. 3:e233. 10.1017/ash.2023.503. 38156215 PMC10753470

[wxag050-B47] Zhang X, Jia N, Wang Z. 2020. The relationship between the filtering facepiece respirator fit and the facial anthropometric dimensions among Chinese people. Ind Health. 58:318–324. 10.2486/indhealth.2019-0158. 31787708 PMC7417508

[wxag050-B48] Zhuang Z, Bradtmiller B, Shaffer RE. 2007. New respirator fit test panels representing the current US civilian work force. J Occup Environ Hyg. 4:647–659. 10.1080/15459620701497538. 17613722

