# Supplementary material for: N95 filtering facepiece respirator fit assessment outcomes by gender, age, race, and facial hair in a community population sample
Source: Ann Work Expo Health. 2026 Jun 23;70(5):wxag050. doi: 10.1093/annweh/wxag050 (PMC13289748; doi:10.1093/annweh/wxag050)
Supplement: wxag050_Supplementary_Data [file wxag050_supplementary_data.pdf]

Title: N95 filtering facepiece respirator fit assessment outcomes by gender, age, race, and facial hair in a community population sample

Majid Bagheri Hosseinabadi[1]

Minji Yu[2]

Ashley Petersen[4]

Linsey Griffin[3]

William Durfee[4]

Susan Arnold[1,*]

[1] Division of Environmental Health Sciences, School of Public Health, University of Minnesota, Minneapolis, MN, USA. ORCID ID: https://orcid.org/0000-0002-5477-199X

[2] Department of Clothing and Textiles, College of Human Ecology, Yonsei University, Seoul, Korea. ORCID ID: https://orcid.org/0000-0003-3997-6409

[3] College of Design, University of Minnesota, 1985 Buford Avenue, 240 McNeal Hall, Saint Paul, MN 55108, USA. ORCID ID: https://orcid.org/0000-0003-4058-5106

[3] Division of Biostatistics and Health Data Science, School of Public Health, University of Minnesota, Minneapolis, MN, USA. ORCID ID: https://orcid.org/0000-0001-7711-9657

[4] Department of Mechanical Engineering, College of Science and Engineering, University of Minnesota, Minneapolis, MN, USA.

[*] Corresponding Author: School of Public Health, University of Minnesota, 1226 Mayo Memorial Building, 420 Delaware St. SE, Mayo Mail Code 807, Minneapolis MN 55455, USA. Email: arnol353@umn.edu ORCID ID: https://orcid.org/0000-0002-1465-3761

Supplemental Material

Table S1: The distribution of the fit factor scores across gender, age group, race/ethnicity, and type of facial hair

Category	N95 Respirator Fit Factor Range, n (%)						
	<25	25-49	50-74	75-99	100-124	125-149	≥150
**Gender**							
Female	19 (7.8)	28 (11.4)	30 (12.2)	45 (18.4)	51 (20.8)	25 (10.2)	47 (19.2)
Male with Facial Hair	19 (26.8)	14 (19.7)	19 (26.8)	8 (11.3)	5 (7.0)	0	6 (8.5)
Male without Facial Hair	7 (11.3)	11 (17.7)	10 (16.1)	11 (17.7)	9 (14.5)	5 (8.1)	9 (14.5)
Non-Binary	0	0	1 (16.7)	3 (50.0)	1 (16.7)	0	1 (16.7)
**Age Group**							
≤19	2 (3.3)	4 (6.7)	9 (15.0)	11 (18.3)	11 (18.3)	5 (8.3)	18 (30.0)
20-29	9 (20.9)	4 (9.3)	6 (14.0)	6 (14.0)	9 (20.9)	5 (11.6)	4 (9.3)
30-39	4 (9.8)	9 (22.0)	6 (14.6)	11 (26.8)	4 (9.8)	2 (4.9)	5 (12.2)
40-49	7 (17.1)	8 (19.5)	4 (9.8)	8 (19.5)	7 (17.1)	2 (4.9)	5 (12.2)
50-59	7 (10.9)	8 (12.5)	7 (10.9)	12 (18.8)	14 (21.9)	8 (12.5)	8 (12.5)
60-69	7 (8.8)	12 (15.0)	20 (25.0)	9 (11.3)	13 (16.3)	7 (8.8)	12 (15.0)
70+	9 (16.4)	8 (14.5)	8 (14.5)	10 (18.2)	8 (14.5)	1 (1.8)	11 (20.0)
**Race and Ethnicity**							
White	37 (12.0)	44 (14.3)	44 (14.3)	56 (18.2)	56 (18.2)	22 (7.1)	49 (15.9)
American Indian or Alaskan Native	0	2 (13.3)	1 (6.7)	4 (26.7)	4 (26.7)	2 (13.3)	2 (13.3)

Asian	4 (14.8)	5 (18.5)	5 (18.5)	2 (7.4)	3 (11.1)	3 (11.1)	5 (18.5)
Black or African American	0	1 (8.3)	5 (41.7)	2 (16.7)	0	2 (16.7)	2 (16.7)
Hispanic or Latino	3 (18.8)	1 (6.3)	3 (18.8)	2 (12.5)	1 (6.3)	1 (6.3)	5 (31.3)
Other	1 (16.7)	0	2 (33.3)	1 (16.7)	2 (33.3)	0	0
**Facial Hair (Male with Facial Hair Only)**							
Full Beard	13 (52.0)	6 (24.0)	5 (20.0)	1 (4.0)	0	0	0
Goatee	2 (18.2)	3 (27.3)	2 (18.2)	3 (27.3)	0	0	1 (9.1)
Mustache	1 (12.5)	1 (12.5)	3 (37.5)	1 (12.5)	0	0	2 (25.0)
Stubble	3 (11.1)	4 (14.8)	9 (33.3)	3 (11.1)	5 (18.5)	0	3 (11.1)

1
